# Picture This: Enhancing Biology Education through Artistic Expression

**DOI:** 10.1093/iob/obag029

**Published:** 2026-06-22

**Authors:** S E Barnes, M R Maxwell

**Affiliations:** Department of Health Sciences, Boston University, Boston, MA 02215, USA; Department of Mathematics and Natural Sciences, National University, San Diego, CA 92123, USA

## Abstract

In this perspective, we describe the Biology through Art project, an interdisciplinary approach to biology education that fosters creativity, engagement, and innovation through the inclusion of visual arts. Decades of research highlight the intrinsic connection between scientific practice and visual representation, underscoring drawing as a critical tool for hypothesis generation, experimental design, data visualization, and scientific communication. Yet, despite these demonstrated cognitive and academic benefits, art-based approaches remain underutilized in undergraduate biology, often due to student anxiety about drawing proficiency, instructor unfamiliarity with art pedagogy, time constraints, and challenges to scaling assignments to large classes. We discuss solutions to these barriers, and present recent examples of course assignments that enhance biology instruction through artistic expression. These assignments, developed for organismal and integrative biology courses by Biology through Art faculty, stimulate active learning, promote conceptual synthesis, and enhance scientific literacy. By presenting research-supported benefits, addressing barriers, offering adaptive curricular models, and highlighting collaborative strategies, we encourage biology educators to integrate art inclusion as a pathway to cultivate scientifically literate, creative, and engaged biology learners.

## Introduction

This perspective describes an active, ongoing project centered on the interdisciplinary teaching of biology: Biology through Art. The Biology through Art project is a collaboration of biologists and artists that promotes artistic expression in biology courses with the goal of fostering creativity, engagement, and long-lasting learning among students. As data collection to evaluate these goals is ongoing, we speak to the project’s rationale and conceptual themes, in addition to offering example assignments and strategies for addressing common barriers to art inclusion in biology courses.

The Biology through Art project finds motivation in calls for interdisciplinary approaches to science education, with national advisory bodies urging integration of the arts and humanities into science, technology, engineering, and mathematics (STEM) curricula in recent years (American Association for the Advancement of Science 2011; National Academies of Sciences, Engineering, and Medicine 2021). Scientists and philosophers alike advocate for an interdisciplinary education of the biology student as a way to promote creativity and new discovery (King 1989; Loehle 1990; Moore 1993; Lerner 2007; Bly 2010; Andreasen and Ramchandran 2012; Gurnon et al. 2013; Bozzone and Doyle 2017). In the classroom, interdisciplinary coursework promotes comprehension of concepts and course engagement (Husband 2015; Murrant et al. 2015; Hymers and Newton 2019). More specifically, integrating the visual arts into biology is quite natural, as drawing, sketching, and diagramming are central to generating hypotheses and models, designing experiments, visualizing data, connecting form and function, and communicating results (Schwarz et al. 2009; Ainsworth et al. 2011; Quillin and Thomas 2015).

The education literature demonstrates diverse cognitive and academic benefits through engagement in relatively “low-tech” art forms, such as sketching and drawing. These benefits include improved attention, memory, observational skills, self-assessment, and organization and synthesis of ideas (Fiske 1999; Deasy 2002; Andrade 2010; Bobek and Tversky 2016; Hardiman et al. 2014; Van Meter and Garner 2005; Jee et al. 2014; Peppler et al. 2014; Quillin and Thomas 2015; Polfus et al. 2017; Meade et al. 2018; Hardiman et al. 2019). Sketching concepts and drawing organisms promote active engagement in course activities by students and contribute to their development of scientific literacy (Merkle et al. 2020; Bal 2023; Flores et al. 2023; Higley et al. 2024). Importantly, drawing engages motoric, pictorial, and elaborative cognitive processes more deeply than passive viewing or verbal description (Ainsworth et al. 2011; Wammes et al. 2016). Student drawings also reveal mental models that can guide conceptual change and model-based reasoning (Quillin and Thomas 2015; Rasmussen and Grinath 2025).

In terms of results seen in the classroom, several empirical studies show benefits of including drawing in biology courses at the university level. These benefits include increased attention to detail (Stagg and Verde 2019), higher exam scores (Chaudhuri 2021), increased self-efficacy (Baldwin and Crawford 2010), and increased appreciation of nature (Merkle et al. 2020). Artwork is analogous to laboratory activities in that both promote active learning, development of manual skills, inquiry, exploration, and engagement among students (Ainsworth et al. 2011). Designing and creating an art piece requires a student’s mental and manual faculties, as the student is tasked to interpret course material, plan out a visual display, and produce a physical piece. The process of art creation generates student–instructor interaction through initial discussions about starting or composing the art piece, as well as follow-up discussions about the progress of the piece. In addition, art creation fosters student–student interaction and coaching, as students often exchange pointers, advice, and critiques during in-class art activities (Higley et al. 2024; Maxwell, personal observations).

Art inclusion supports diverse learning approaches and preferences, such as non-verbal expression and visual depiction of ideas (Quillin and Thomas 2015; Stagg and Verde 2019). Incorporating diverse learning preferences promises to serve students that pursue alternative educational pathways. One such alternative pathway is that of non-traditional students: students older than 18–22 years, often with full-time jobs and dependent family members (Choy 2002; Hussar and Bailey 2011; Soares 2013; Kasworm 2014; Rabourn et al. 2018; Knowles et al. 2020). While the proportion of non-traditional undergraduate students in the USA has increased since 2000 (Choy 2002; Kazis et al. 2007; Soares 2013), teaching strategies developed to serve such students remain relatively unexplored (Wyatt 2011; Freeman et al. 2014; Schinske et al. 2017; Barral et al. 2018; Stains et al. 2018). This is worrisome, given the challenges that non-traditional students face, including lower degree completion rates and difficulties integrating into the university environment (Choy 2002; Taniguchi and Kaufman 2005; Ross-Gordon 2011; Bergman et al. 2014; Miller 2014; Markle 2015; Ellis 2019). Interestingly, Wyatt (2011) notes that, while non-traditional students are less likely to participate in extracurricular activities, such as research with a faculty member or campus-based outreach, they are more engaged while in class. Active learning strategies that promote student engagement with content through applications of ideas and concepts, such as in-class discussions, group work, and artistic expression (Meyers and Jones 1993; Rabourn et al. 2018; Driessen et al. 2020; Lombardi and Shipley 2021), are therefore expected to be particularly effective with non-traditional, especially those above 30 years of age, often termed “adult students” (Rabourn et al. 2018; Knowles et al. 2020). In addition to promoting active engagement with course material, artwork allows for individual expression of course concepts. The opportunity for some degree of autonomy and choice over the learning process is expected to reinforce active engagement by adult learners (Knowles et al. 2020). More generally, incorporating self-discovery into the learning process leads to greater retention and scholastic motivation among all students (Matern and Feliciano 2000).

## The Biology through Art project

Motivated by the benefits of art inclusion in the biology classroom, the Biology through Art project brings together over 70 biologists and artists across 50 organizations in the USA and Canada to collaborate on enriching biology courses with the visual arts. The project was formally established in 2021 through funding by the National Science Foundation’s Research Coordination Networks in Undergraduate Biology Education (RCN-UBE). Biology through Art continues as a funded project through NSF’s Improving Undergraduate STEM Education program (IUSE). Notable project participation occurs throughout North America, including the West Coast (e.g., National University, California State University Fullerton, San Diego Mesa College), Midwest (e.g., Northeastern Illinois University, Eastern Illinois University), and East Coast (e.g., North Carolina State University, Boston University, University of North Florida, Emory University). Collectively, these schools serve urban and rural populations, under-represented groups, non-traditional, and active and veteran military students.

Since 2021, Biology through Art has supported the integration of artwork in 78 courses in participating colleges and universities, including lower and upper division courses and courses for majors and non-majors. In recent years (2023–26), the project has focused on upper division courses to test the efficacy of art inclusion with advanced topics in biology; to date, 43 upper division courses form this ongoing study (Table 1). Support for art inclusion involves regular meetings among project members to share ideas on designing and evaluating art-based activities and assignments, and on collecting data on student performance and engagement. The project promotes teacher development by coaching and mentoring biology instructors on introducing art activities and assignments on a trial or exploratory basis. Experienced project members and artists visit classrooms to guide such activities, when feasible. Annual workshops serve as additional opportunities for teacher development, and for interactions among project biologists and artists. Through these efforts, the Biology through Art project seeks to build instructor competence and confidence with respect to art inclusion in their courses.

**Table 1:** Scope of the Biology through Art project: participating universities and upper division biology courses in three academic years (July 2023–June 2026, 43 courses).

University	# Courses	Course titles	Enrollment per course (range)
Boston University	3	Immunopathology; Historical and Physiological Perspectives on Organ Transplantation	23–45
California State University Fullerton	11	Comparative Animal Physiology, Human Physiology, Vertebrate Biology	14–96
Eastern Illinois University	2	Animal Behavior	6–16
Emory University	1	Visual Communication in Biology	21
National University	10	Animal Behavior, Cell Biology, Ecology, Invertebrate Zoology	5–20
North Carolina State University	3	Animal Diversity	120
Northeastern Illinois University	3	Local Fauna, Ornithology	18–24
University of North Florida	3	Plant Anatomy & Physiology, Scientific Illustration	16–43
Virginia Commonwealth University	7	Coastal Ecology, Comparative Vertebrate Anatomy, Evolution of Angiosperms, Human Metabolic Disorders	14–41

Biology through Art’s goals extend to student development and public outreach. The project organizes regular student art exhibitions. These exhibitions are in the format of poster sessions at scientific conferences, in which students entertain questions and discussion about their art pieces. The exhibitions are typically open to the public and held at museums and libraries such as the San Diego Natural History Museum, Museum of Contemporary Art San Diego, and the Bonita Museum and Cultural Center. Additionally, project biologists and artists present art-inclusion activities at local STEM and science, technology, engineering, arts, and mathematics (STEAM) festivals, such as San Diego’s Festival of Science and Engineering, which draws over 15,000 visitors per year. Particularly effective outreach activities are those that harmonize concepts in biology and art, such as color, symmetry, and repetition of visual motifs, through stencil drawings, puzzles, and matching games.

## Overcoming barriers to including art in the biology classroom

Although the benefits of art inclusion are well documented, instructors and students often perceive barriers that limit widespread adoption. These barriers are multifaceted, as revealed through both the literature and faculty experiences within the Biology through Art project. Here, we address these barriers and provide recommendations for overcoming them.

A common refrain among students encountering drawing assignments is “I’m not good at drawing,” which often manifests as anxiety or avoidance of art-based tasks (Quillin and Thomas 2015; Merkle et al. 2020; Flores et al. 2023). This lack of confidence can severely limit student engagement with drawing activities. Misconceptions that artistic talent or prior training is necessary exacerbate such anxiety. Notably, even in courses that emphasize observational drawing, students may initially fail to recognize its value as a scientific process and view it as peripheral to biology content (Quillin and Thomas 2015; Merkle et al. 2020; Flores et al. 2023). Therefore, clear communication should foster an environment that normalizes imperfection, emphasizes that artistic skill is not a prerequisite, and reinforces drawing as a process of sense-making and scientific observation (Quillin and Thomas 2015; Beavington 2016; Merkle et al. 2020; Flores et al. 2023; Higley et al. 2024). Scaffolded instruction in which the instructor introduces relevant drawing techniques accompanied by examples, exercises, and iterative feedback supports student confidence development and improves learning efficacy (Merkle et al. 2020; Rasmussen and Grinath 2025). Creating clear and transparent grading criteria may also reassure students of how they will be assessed.

In a recent survey among faculty participating in the Biology through Art project, faculty reported that structural factors such as large class sizes and limited class time posed significant challenges to incorporating drawing and other art-based assignments (unpublished data). Time constraints emanate both from the breadth of required biology content (“tyranny of content”) and the added demand of dedicating time to teaching, practicing, and assessing art activities (Rasmussen and Grinath 2025). However, Biology through Art faculty affirmed that grading art assignments required comparable or sometimes less time than grading writing assignments. Strategically integrating drawing into existing curricular modules or discussion sections, particularly in large-enrollment courses, may help to alleviate these challenges (Beavington 2016). Furthermore, taking advantage of peer- and self-assessment may not only reduce grading burden, but may also promote student metacognition and engagement in the artistic and scientific processes (Rasmussen and Grinath 2025). In addition to time constraints and difficulty in scaling assignments to large class sizes, lack of access to suitable teaching spaces and art supplies may deter some educators from integrating art assignments into their curricula. To mitigate these logistical challenges, Biology through Art faculty and others have frequently utilized low-cost materials such as pencils and paper (Stagg and Verde 2019; Rasmussen and Grinath 2025). A minimal pencil-and-paper approach may be most appropriate for large lecture classes, where focus is placed on diagramming concepts, sketching anatomy, or drawing multi-panel cartoons to illustrate biological processes. Such activities can be done in large auditoriums in the forms of short assignments worth nominal points or as brief sketch-and-compare discussions among students. Practical strategies and resourceful approaches developed by Biology through Art faculty therefore demonstrate that effective integration of art-based assignments into biology curricula is both feasible and beneficial.

While Biology through Art faculty generally reported confidence in grading art-based assignments, concerns remain around creating clear grading criteria to reduce subjectivity, optimize grading efficiency, and provide clear instructions to students on how to succeed. These concerns can deter instructors, especially those lacking previous art instruction experience, from adopting drawing assignments (Flores et al. 2023). Faculty express the need for shared resources, including sample rubrics, lesson plans, and assignment examples to support implementation across diverse course contexts. Moreover, differentiating the learning benefits of art inclusion per se from time-on-task effects remains a methodological challenge that can influence faculty buy-in. To support instructors, expanded dissemination of sample lesson plans, rubrics, and interdisciplinary collaboration between art and biology faculty—as cultivated by the Biology through Art network—can build instructor competence and confidence in facilitation and assessment.

## Artwork in the biology classroom

In this section, we present three activities developed by Biology through Art faculty at National University and Boston University that illustrate practical approaches for integrating artistic expression into undergraduate biology courses. Designed for organismal and integrative biology courses, these activities highlight how targeted drawing and visualization assignments can support key pedagogical goals such as enhancing observational skills, fostering conceptual synthesis, and promoting student engagement. Drawing on instructional materials, student work, and assessment strategies developed through the Biology through Art project, these examples provide adaptable frameworks and resources for faculty seeking to incorporate art-based learning into diverse biology course contexts, including activities that require relatively short class time (Assignment 1) to iterative, course-long projects involving graphical abstracts (Assignment 2) and acrylic paintings (Assignment 3).

### Assignment 1: Drawing an organism

A concise, focused assignment is drawing an organism. The goals of such an assignment typically include fostering observation, attention to detail, and expression and description of anatomy and morphology. Drawings of organisms are clearly relevant to organismal courses, such as Vertebrate Zoology, Ornithology, Invertebrate Zoology, Entomology, and Plant Biology. Drawings of organ systems can be incorporated in Comparative Anatomy, and drawings of cells and cellular structures can form part of Cell Biology, Microbiology, and Molecular Biology. The following discussion offers a general approach that can be adopted to these diverse courses. An accompanying lesson plan with pointers to instructors, a list of drawing materials, and a grading rubric is provided in Appendix 1.

The appeal of drawings lies in relatively minimal start-up costs: pencil, paper, and eraser. The subject organisms can be photos or drawings (hard copies or electronic images), three-dimensional models, laboratory specimens, or organisms seen during a field trip. For drawings in the field, plant parts, such as leaves or flowers, serve as useful models for training students to notice and express anatomical details.

**Fig. 1:**
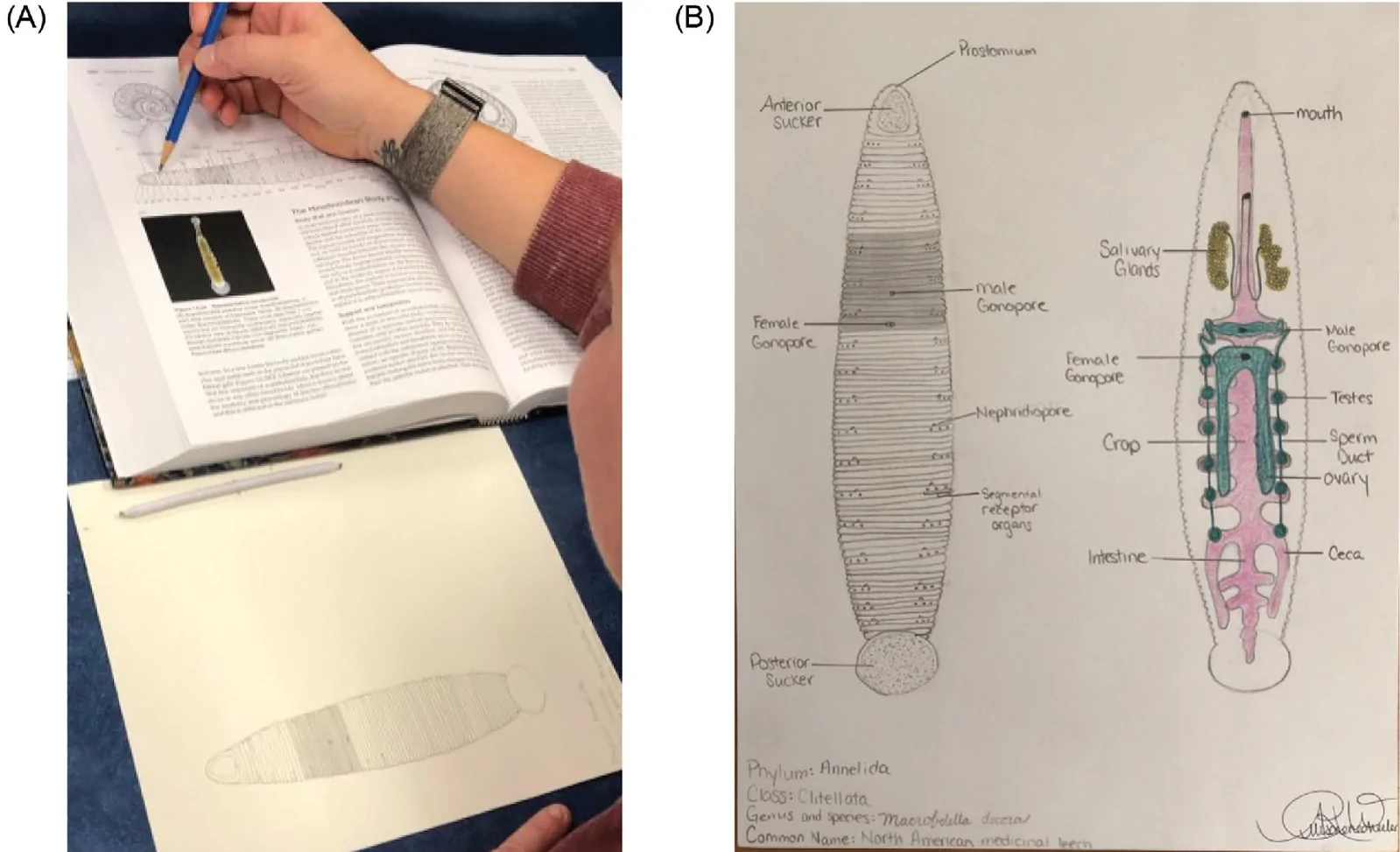
Drawing of the anatomy of the North American medicinal leech (*Macrobdella decora*). (A) Verifying external anatomy in initial stages of drawing. (B) Finished drawing, demonstrating attention to anatomical details. On left, graphite pencil; on right, graphite and colored pencils. (Drawing by student A. Wheeler, National University; images used with permission. Photo credits: M.R. Maxwell.)

To address barriers regarding supplies and student perceptions of their drawing ability, students can be provided with drawing materials (e.g., pencils, erasers, sharpeners, and blending stumps) and should be coached by the instructor in the early stages of the assignment. Students can be allowed to begin their drawings during class time, such as during discussion or laboratory periods (Fig. 1A). Biology through Art faculty find that 20 min is often enough for students to become invested and generate momentum towards completing the drawings as homework (Fig. 1B).

**Fig. 2:**
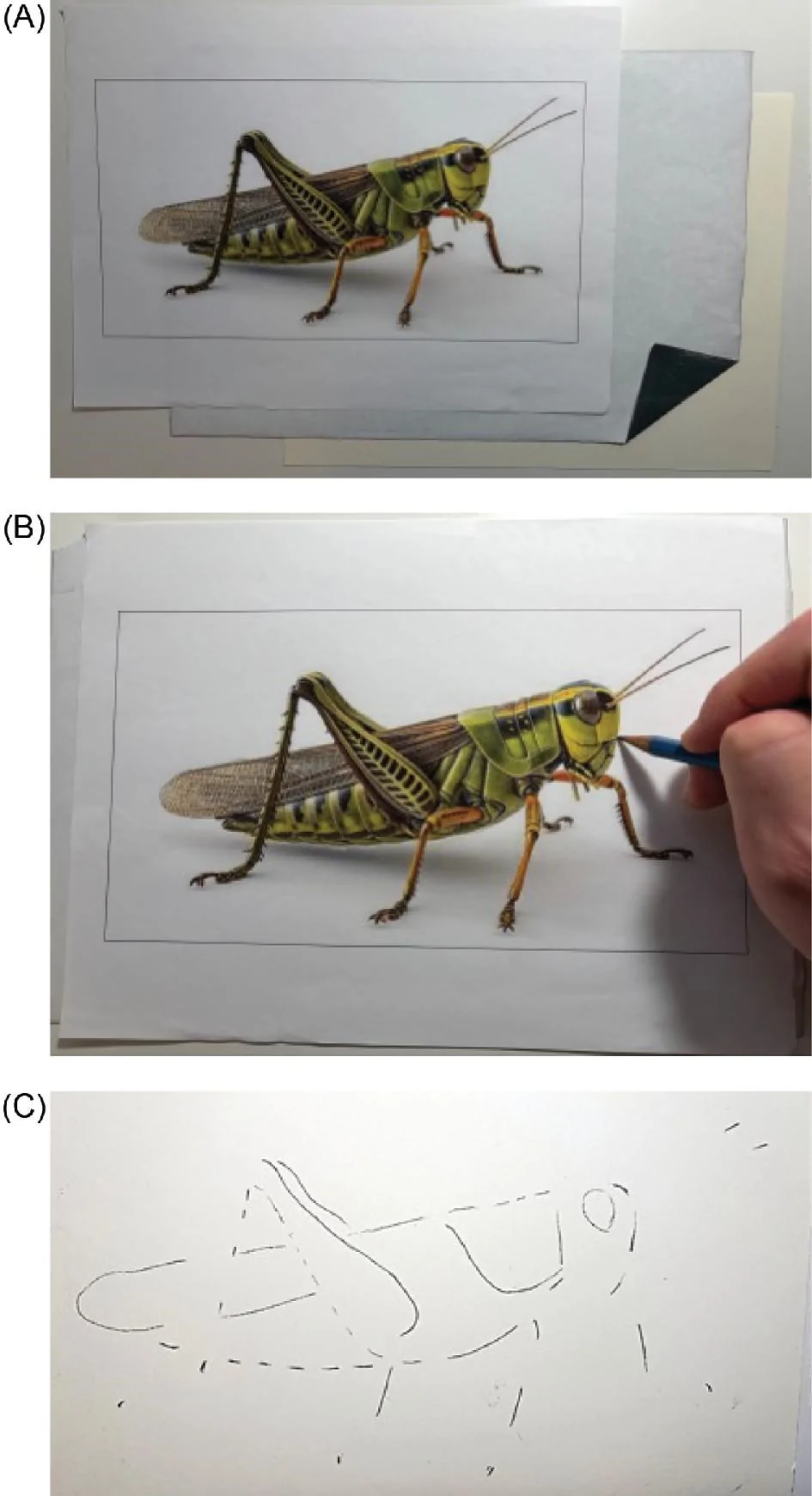
Starting a drawing with the use of carbon transfer paper. (A) Arrangement of model image on top, transfer paper in middle (carbon facing down), drawing paper on bottom. (B) Tracing main body regions, structures, and contours. (C) Carbon impressions outline the body of a grasshopper. (Tracings by author M.R. Maxwell. Photo credits: M.R. Maxwell.)

An effective strategy for building student confidence is to allow for tracing via carbon transfer paper (Fig. 2A). By allowing students to map out an organism’s overall proportions and morphology, the students identify main body regions and structures (Fig. 2B–C). Once these “landmark” body parts have been traced, students are typically able to complete the drawings free hand.

**Fig. 3:**
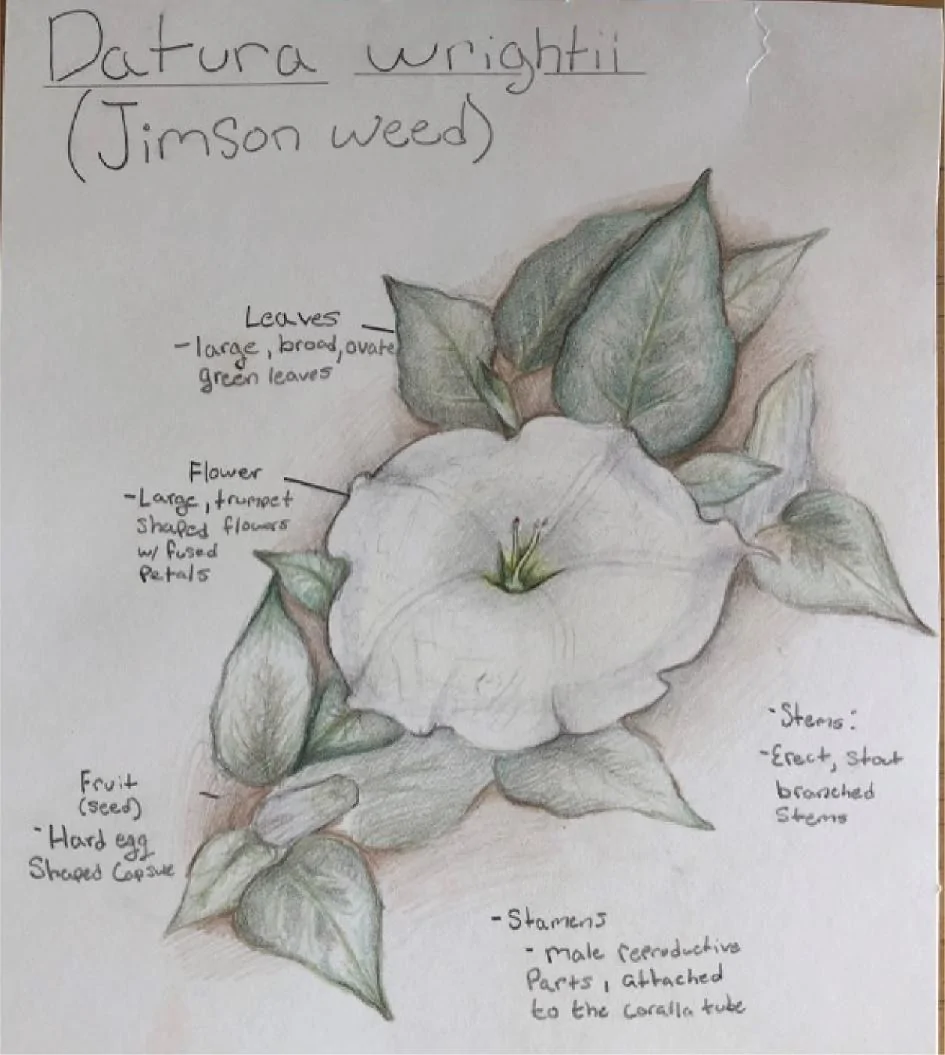
Drawing of jimson weed (*Datura wrightii)*; graphite and colored pencils. (Drawing by student J. Moreno, National University; image used with permission. Photo credit: M.R. Maxwell.)

Grading rubrics should emphasize proper body proportions of organisms, correct position and form of body structures, and labels or annotations of structures (Figs. 2, 3; Appendix 1). Instructors may want to assign a small number of points for “aesthetics” and “effort” to reward students’ skill and labor while not setting unrealistic artistic standards in a biology course. The primary focus should be depicting and retaining knowledge of organismal biology, with drawing being the conduit of student expression.

### Assignment 2: Integrating physiological mechanisms through course-long sketches and a graphical abstract

In the courses Immunopathology (SARHS 455) and Historical and Physiological Perspectives on Organ Transplantation (SARHS 465) at Boston University, students were taught the mechanisms underlying a variety of immune responses. To date, three student cohorts have completed this iterative project (*n* = 24–45 students per cohort). Students were upper division undergraduate or master’s students majoring in Biology, Human Physiology, Health Science, and/or Biomedical Engineering. This description summarizes the assignment framework rather than providing data for any specific cohort.

In both courses, a series of lectures were devoted to the principles of immunology, with additional lectures focusing on specific types of immune responses. Following each lecture, students were provided prompts to integrate content from that lecture into a visualization (“sketches”). At the end of SARHS 465, students synthesized their sketches into a publishable graphical abstract. The objective of these assignments was for students to synthesize their understanding of the lecture, organize ideas, and integrate content from that lecture with previous lectures.

**Fig. 4:**
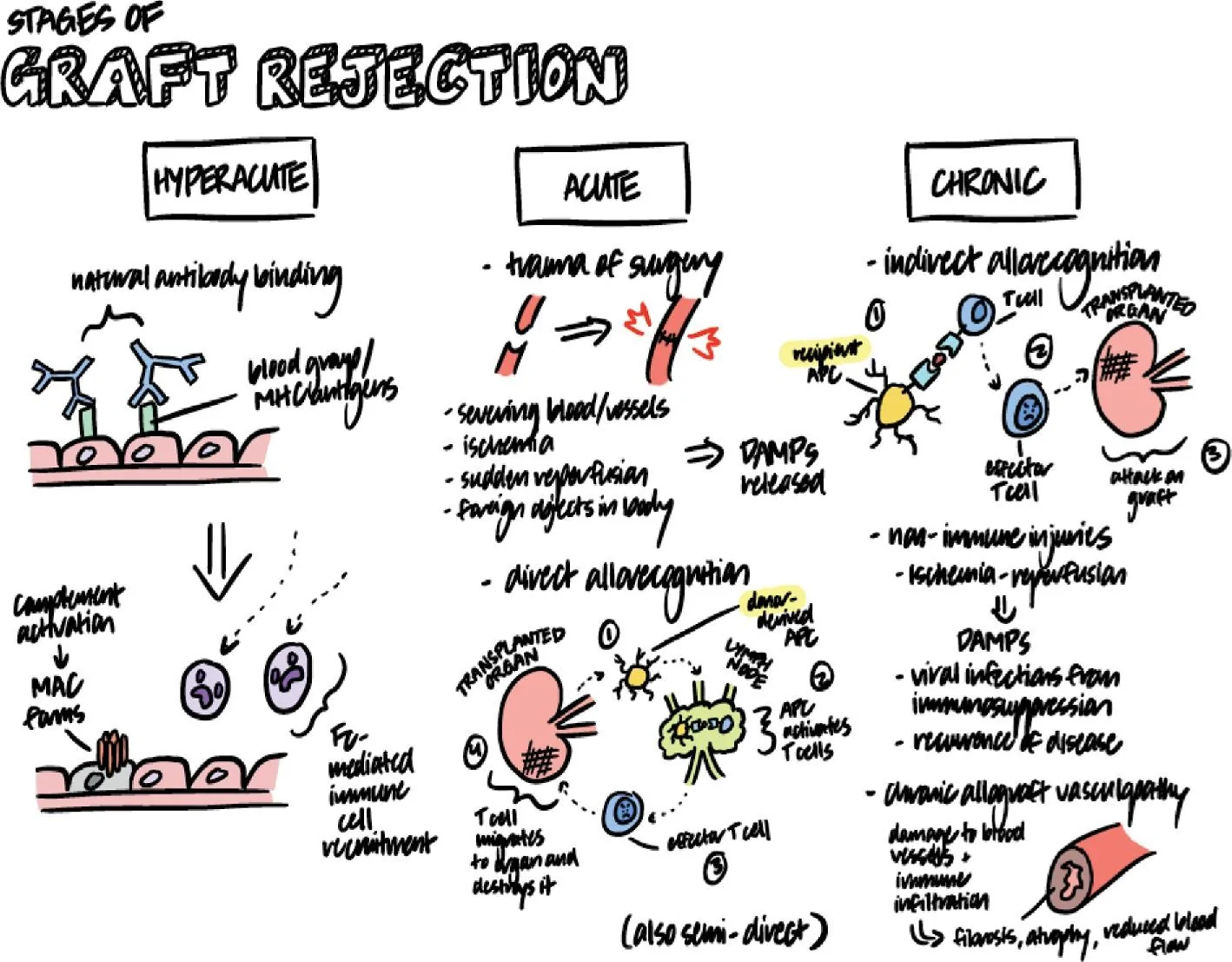
Student sketch depicting the general stages of organ transplant rejection. Following an introductory lecture on organ transplant rejection, the student sketched mechanisms for hyperacute, acute, and chronic graft rejection. The sketch reveals the student’s artistic expression and engagement through anthropomorphism of immune cells such as frowning faces on anti-graft effector cells. (Sketch by student L. Okimoto, Boston University; image used with permission.)

In the first lecture of each course, 30 min were committed to providing instruction on various approaches to visually representing scientific knowledge including concept maps, flowcharts, diagrams, and illustrations. Students also examined how various methods of visualization were integrated to create graphical abstracts. This lecture included examples of various types of visual representation. It was also emphasized that visual representation is an important skill in the scientific process that would benefit them in both their academic and professional careers. In each lecture, students were prompted to visually integrate content from that lecture and synthesize it with content from previous lectures. These prompts included the form that the visualization should take (systems diagram, illustration, etc.), as well as all of the content that needed to be depicted in the visualization. For example, in one sketch, students illustrated the general stages of graft rejection (Fig. 4). In the next sketch, they applied these stages to a specific organ of their choice. In the third, they added targets of immunosuppression. While 5–10 min was provided at the end of class to work on sketches, they were mostly completed as homework. At the beginning of the following lecture, students peer reviewed each other’s sketches before coming together as a class to discuss challenges that students faced and potential solutions to those challenges. Roughly 5–10 min were dedicated to discussion at the beginning of each lecture.

**Fig. 5:**
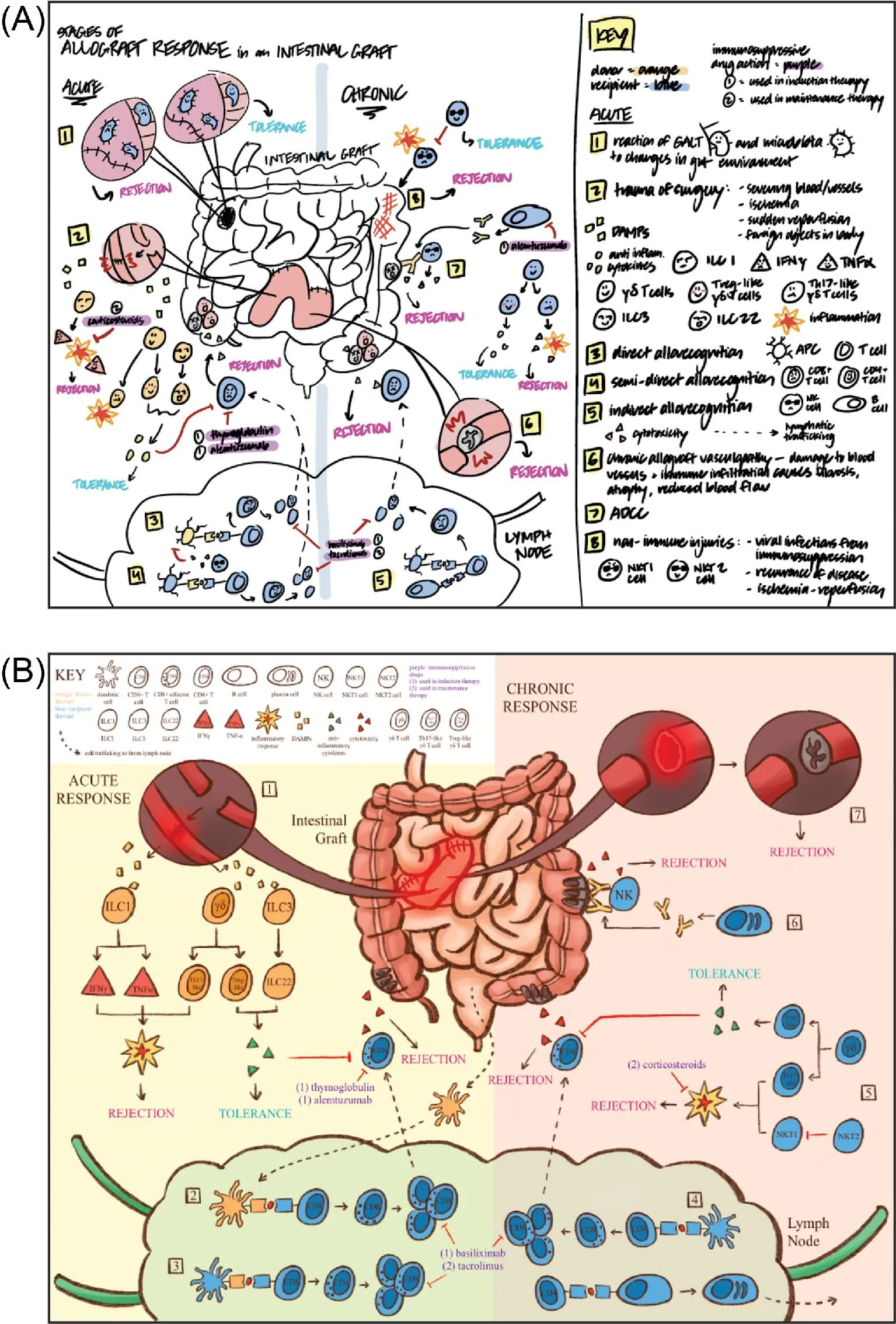
Student’s rough and final drafts of a graphical abstract depicting intestinal transplant rejection. Students were instructed to use the sketches they had created throughout the course (Fig. 4) as the basis for the rough (A) and final (B) drafts of their graphical abstract. For the final graphical abstract, students were required to include all major cell types and their mediators/mechanisms of action with clear distinctions on which cells are donor- versus recipient-derived. The figure needed to be professional, aesthetically pleasing, publishable, thorough, organized, and easy to read. (Graphical abstracts by student L. Okimoto, Boston University; image used with permission.)

Finally, students in SARHS 465 adapted their sketches into a publishable graphical abstract (Fig. 5). Students were allowed to use any media that they preferred as long as the content was clear and legible. Most commonly, students drew their illustrations on a self-provided tablet. Other students used pencil and paper, a whiteboard, or programs such as Microsoft PowerPoint and Adobe Illustrator. For the final graphical abstract, many students used a free version of BioRender, though some students still opted to illustrate their own images. In this regard, the assignments were low-cost, accessible to students, and promoted individual expression.

Clear grading rubrics for both the sketches and final graphical abstract were provided at the beginning of the semester to reduce student anxiety and facilitate objective and efficient grading. Sketches were formative assignments that received full credit as long as they were in the appropriate form and contained all the required content. While artistic ability was not included in the grading rubric, many students did utilize these assignments as an opportunity for artistic expression (Fig. 4). The final graphical abstract was evaluated on both form and content. Full credit on the “presentation” criteria was granted to assignments that were “professional, aesthetically pleasing, and publishable,” as well as “thorough, yet organized and easy to read.” The instructor provided feedback on both form and content, and students peer-reviewed each other’s work in class.

Anonymous university-administered course evaluations were submitted at the end of the semester. While one student requested “less sketches please” in the evaluations, every other comment was overwhelmingly positive. Students regularly described the sketches as engaging, with one evaluation stating that “homework was designed to be as enjoyable and low stress as possible” and “sketches were an interesting way to interact with the information.” Students also appreciated how the sketches helped them integrate concepts from multiple lectures into a broad framework, saying “the most valuable aspects were the sketches being almost a study guide for the material” and “the graphical abstracts I felt helped me understand the material so much better.” Of the main skills and knowledge that students obtained during the courses, many students reported developing new artistic skills that they could use while studying and later in their professional careers. One student said the sketches “helped facilitate my individual learning of the material outside of the classroom setting,” and another said they became “more creative with my studying strategies. . . using sketches to study, whiteboarding, drawing.” One student directly stated that “using BioRender and free-draw software to illustrate biological interactions is a great skill I was given from this course and I plan to use this in further educational and professional settings.”

### Assignment 3: Painting animal morphology and phylogenetic relationships

In the course Invertebrate Zoology (BIO 414) at National University, students are tasked to depict the relatedness of 16 major phyla within Kingdom Animalia as an acrylic painting (water-based). To date, five student cohorts have completed this assignment (*n* = 8–16 students per cohort). This description summarizes the assignment, rather than presenting results for a specific cohort. This assignment has a realistic component in that a representative species of each phylum is to be shown with realistic anatomy and morphology. Students introduce an imaginative element in terms of the painting’s setting or context, such as an underwater scene, a forest, or stars that form a galaxy.

The painting process is iterative and spans the entire course. Students are provided painting materials (canvas, brushes, and acrylic paints) and are coached by the instructor in the early stages of the assignment. Students are shown examples from previous cohorts to help them visualize the finished product. In-class time is devoted to working on the painting, during which students conceptualize their paintings, plan them out, and complete them. In-class painting amounts to 1–2 h per week, with 4.7 h of total instruction per week over eight weeks. In-class work does much to alleviate student anxiety over producing a painting, both in terms of time-to-completion and mentoring less confident students. The process of depicting the phylogeny of animal phyla is emphasized over the aesthetics of the final product, with students being assured “try your best, and you’ll surprise yourselves in a positive way.”

**Fig. 6:**
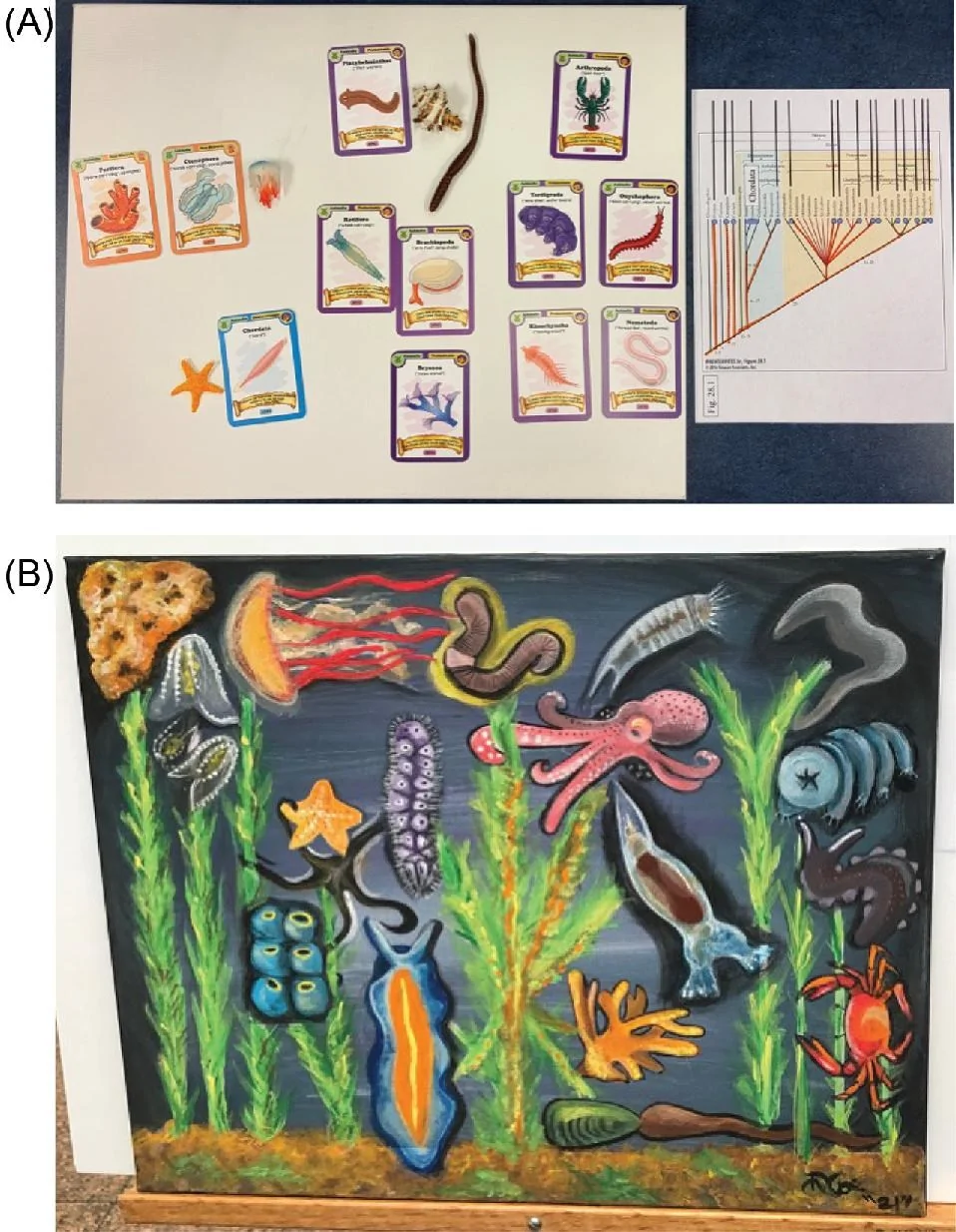
Painting of phylogenetic relationships among major phyla within Kingdom Animalia. (A) Initial grouping of representations of the phyla on a canvas. (B) Finished painting (acrylic on canvas), showing major clades as kelp strands in an imaginative underwater scene. (Painting by student D. Castillo, National University; image used with permission. Photo credits: M.R. Maxwell.)

Student progression through the painting begins with grouping the animal phyla on the canvas with visual cards, model animals, specimens, or rough sketches (Fig. 6A). Arranging the animals reinforces anatomical similarities among related clades, as well as differences between distant clades. Students then select a representative member of each of the 16 phyla to depict, which allows for expression of individual preferences and perspectives. Students also learn principles of artistic composition, as they must fit their selected animals within the canvas, devote comparable space to each, and clearly show phylogenetic relationships among them (Fig. 6B).

The grading rubric for the painting assignment is provided to students at the beginning of the course. The assignment represents 15% of the student’s course grade. The bulk of the assignment’s points (60%) are reflected in accurately showing the phylogenetic relatedness among the phyla and in depicting each phylum’s representative species. Emphasis is placed on each species showing recognizable features that reflect membership in its phylum. “Aesthetics” earns 6% of points, and “effort” earns 10% of points. A 600-word write-up earns the remaining 24% of points, in which students explain the painting’s underlying biological concepts (phylogenetic relationships) and artistic principles (e.g., student’s use of line, color, and space).

This painting assignment strongly reinforces course material and fosters course engagement. The iterative process of developing the painting in-class generates dialogue between the instructor and students about invertebrate anatomy and physiology, typically recalling recent lectures or pointing to upcoming material. Students become invested in their paintings, seeing them as expressions of their knowledge of zoology as well as reflections of their personal thoughts. Additionally, the students naturally mentor each other in terms of depicting invertebrate anatomy, color choices, and whether something “looks right.”

## Conclusion

Integrating drawing, sketching, and other artistic practices into biology education offers far more than an engaging classroom activity; it provides a powerful, research-supported means of strengthening scientific literacy, deepening conceptual understanding, and fostering creativity. When students visualize biological structure and processes, they engage in the same observational, interpretive, and communicative practices that underlie authentic scientific work. These artistic approaches help students develop heightened observational acuity, enabling them to notice subtle morphological details as well as complex biological relationships that traditional instruction often cannot achieve. By inviting students to express their understanding visually, instructors create opportunities for curiosity, autonomy, creativity, and critical thinking—qualities essential for mastering integrative and organismal biology, where comprehension of complex systems and dynamic processes is paramount. This multifaceted teaching approach not only enriches content mastery, but cultivates a more inclusive and motivating classroom environment, accommodating diverse learning styles and backgrounds.

The Biology through Art project exemplifies a collaborative, interdisciplinary effort to expand art inclusion across multiple institutions, serving a wide range of learners, including non-traditional and underrepresented student groups. The curriculum examples presented here demonstrate practical, adaptable approaches that can be tailored to diverse educational contexts and student populations. We encourage biology instructors to embrace art integration as a robust pedagogical tool that enriches the biology learning experience, supports diverse learners, and cultivates the next generation of scientifically literate and creative thinkers.

## Supplementary Material

obag029_Supplemental_File

## Data Availability

No original data was published in this article.
