# Supplementary material for: Picture This: Enhancing Biology Education through Artistic Expression
Source: Integr Org Biol. 2026 Jun 22;8(1):obag029. doi: 10.1093/iob/obag029 (PMC13352434; doi:10.1093/iob/obag029)
Supplement: obag029_Supplemental_File [file obag029_supplemental_file.docx]

**Appendix 1: Lesson Plan for Drawing Assignment (Assignment 1)**

**Lesson Description**

Each student produces a realistic pencil drawing of an organism (in graphite, on 8x10-inch sheet of drawing paper). This assignment emphasizes observation of anatomy and morphology, and realistic depiction of these observations.

**Intended Courses**

Biology courses that include biodiversity, organismal biology, or anatomy and physiology. Any taxon or group of organisms can be the focus of the drawing. Suitable courses include:

high school biology;

collegiate lower division biology with a biodiversity, organismal, or anatomy/physiology component;

collegiate upper division or graduate-level zoology, entomology, mammalogy, ornithology, botany, plant biology, microbiology, etc.

**Key Topics and Concepts**

1) Observe anatomy and morphology of organisms.

2) Depict anatomy and morphology through drawing.

3) Label salient features of an organism’s anatomy.

4) Develop drawing skills.

5) Appraise cross-over concepts in art and biology, such as proportion, placement of body structures (composition), symmetry, texture, contour, body mass or volume.

**Materials**

| Item | Unit cost * | Cost for 30 students | |  |
| --- | --- | --- | --- | --- |
| Drawing (graphite) pencils. Minimum set per student:  6B (soft, dark)  HB (medium)  4H (hard, light) | $17 for 12 pencils of same type (e.g., 6B) | $153 | 36 pencils 6B, 36 pencils HB, 36 pencils 4H; re-usable | 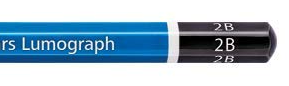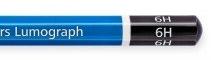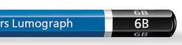 |
| Drawing paper | $8 for 24 sheets (8x10”) | $ 16 | 48 sheets; single-use | 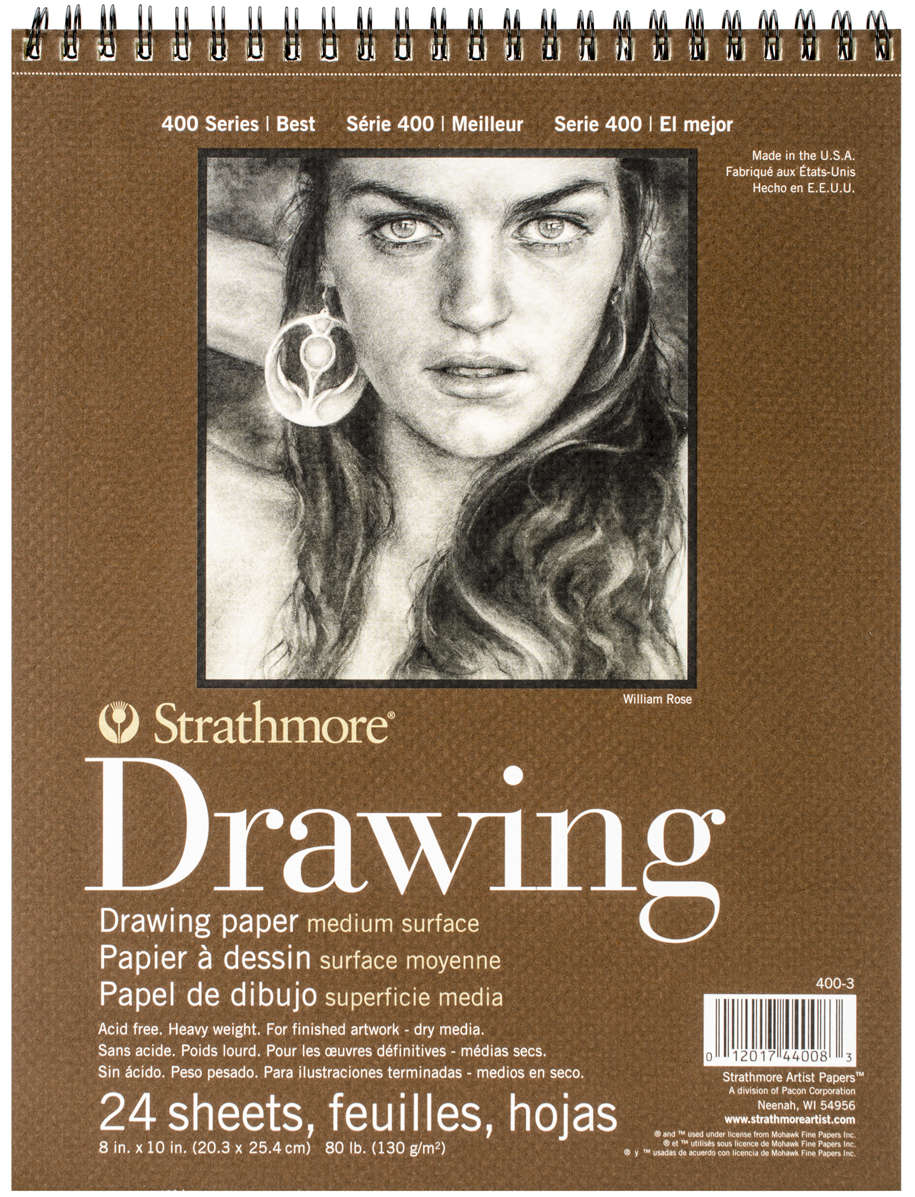 |
| Erasers  Standard (“rub”)  Kneaded | $8 for set | $ 40 | 5 sets, erasers can be cut in half to economize; re-usable | 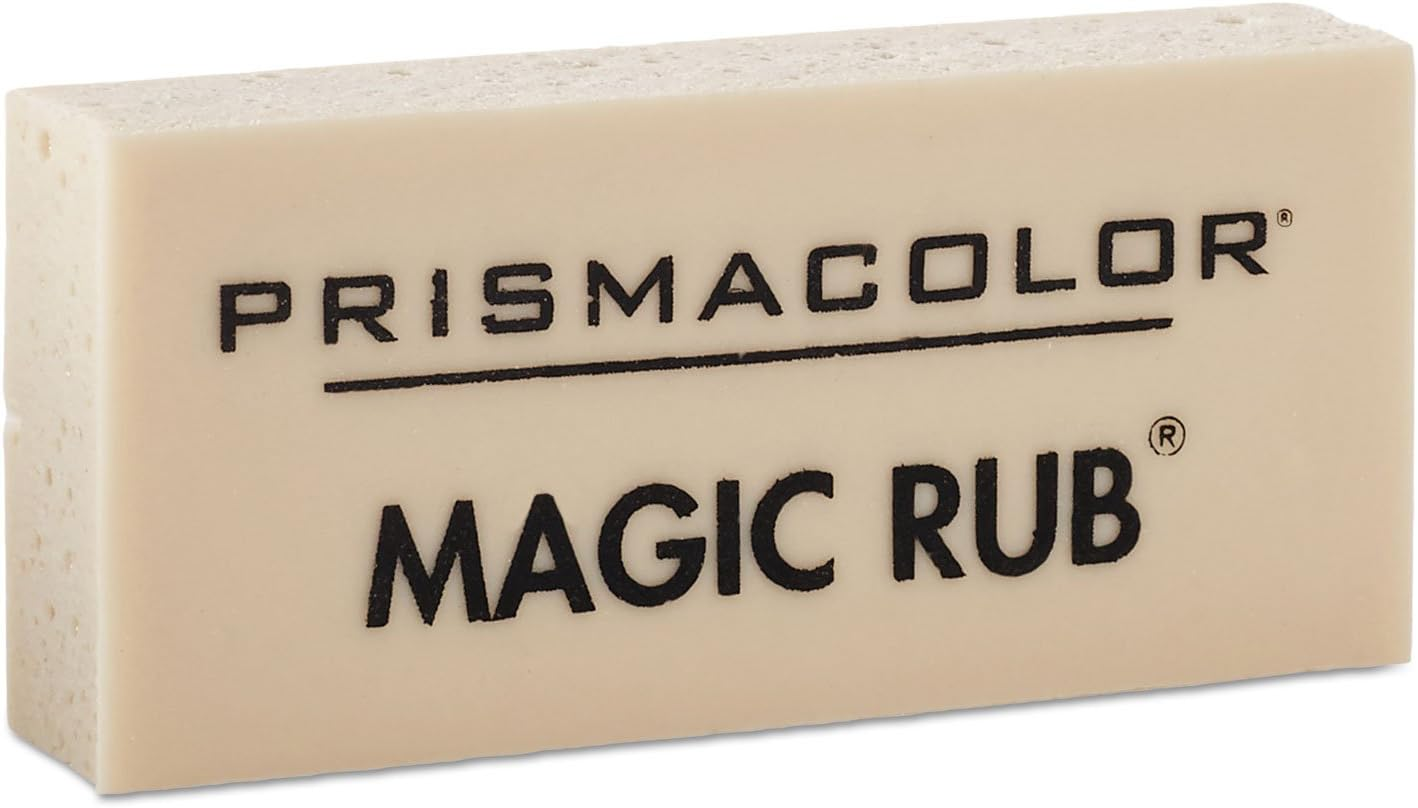 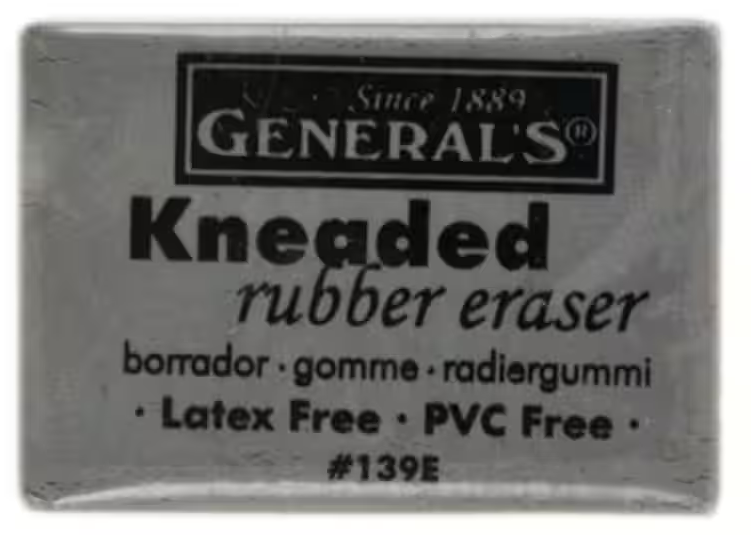 |
| Blending stumps | $12 for 5 sets of 6 stumps each | $ 36 | 15 sets should be sufficient; re-usable | 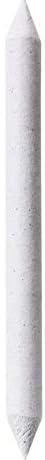 |
| Wedge pencil sharpener | $10 for set of 3 sharpeners | $ 30 | 10 sharpeners should be sufficient; re-usable | 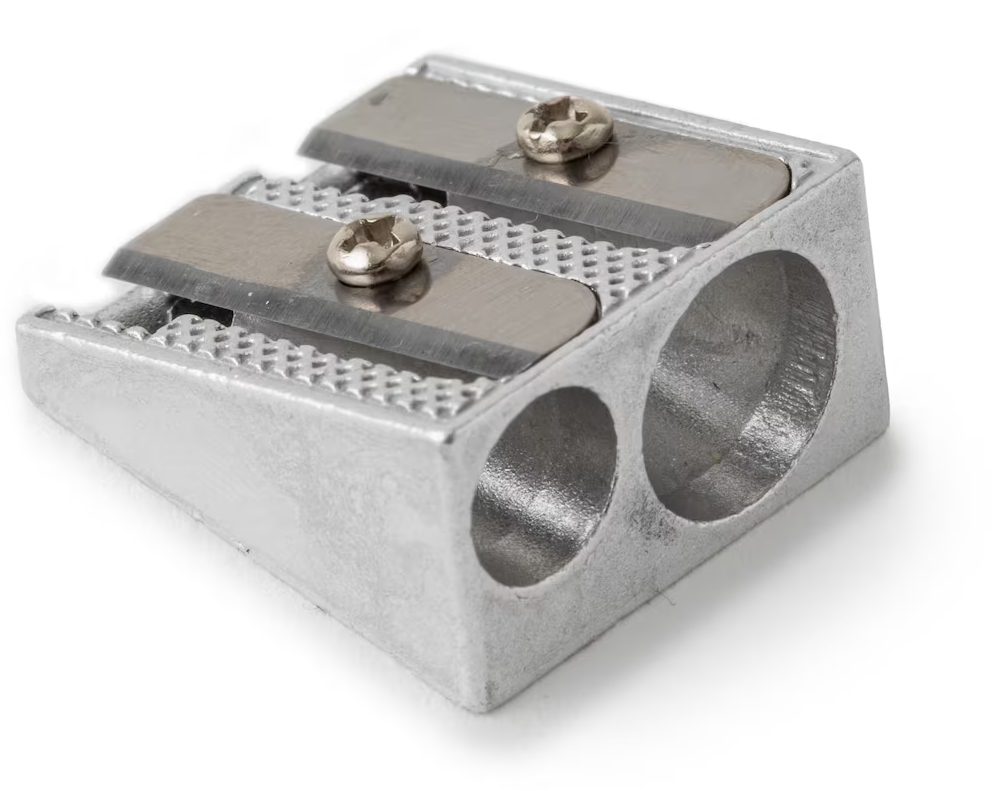 |
| Carbon transfer paper (optional: for tracing) | $5 for 100 sheets | $5 | 100 sheets; re-usable | **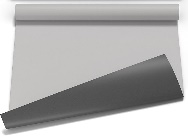** |

* Typical prices as of June 2026, at common art stores or online vendors (e.g., Michael’s, Blick Art Materials).

**Notes to Instructors**

1) This assignment assumes that students have had a previous lesson on drawing basics (30-60 minutes) that includes the use of the different pencil types (e.g., 6B, HB, 6H), the use of blending stumps for blending and smudging, the use of kneaded erasers for lightening graphite marks, and practice in shading and depicting the direction of light. In lieu of presenting such a lesson in this Lesson Plan, novice students can consult useful instructional videos, such as the “Learn to Draw” YouTube series by SchaeferArt (www.youtube.com/@SchaeferArt/playlists).

2) To offset the cost of drawing supplies for students, the instructor may opt to loan communal drawing materials to students for work outside of class time. To encourage the return of the materials, consider assigning a small number of points to these loans. If the student returns all loaned materials, then the student earns these points. This approach has proven to be effective in getting the great majority of loaned materials returned from students.

3) To make the drawing task manageable, especially for novice or beginning drawers, consider allowing students to draw a “floating” organism. That is, the organisms can be drawn in isolation, not grounded in a surrounding habitat or ecological context.

**Lesson Instructions**

1) Drawing of an organism: in graphite, on 8x10-inch sheet of drawing paper. The subject organism can be a photo or drawing (hard copy or electronic image), three-dimensional model, laboratory specimen, or organism seen during a field trip.

2) Total time expected for each student to complete drawing: 2 hours.

3) The illustration should fill most of the drawing space. If the instructor requires labelling of body parts, then a margin of roughly 1 inch should surround the organism.

4) Students typically benefit from starting their drawings during class time, as some students may need guidance on how to begin the drawing, getting initial placement and proportions, etc. Twenty minutes of class time is often sufficient to get students started.

5) To get the students started, instruct them to map out the top-bottom and side-to-side extremities of the organism with light marks (e.g., top of head, end of antennae, end of tail). This will help establish the proportions and placement of main body parts; see **Figure 2.**

6) Tracing (optional). Establishing an organism’s overall proportions and placement of main body parts can be accomplished through tracing with carbon transfer paper **(Figure 2).** Tracing is effective in building student confidence and “buy-in” to the drawing assignment.

7) Once the student has initially placed the organism’s main body parts, the student then applies pencil strokes, using the blending stump to smudge the graphite to show shading and gradients of tone.

8) Completing the drawing is an interplay between capturing the model organism’s body features and details with pencil strokes, and using the blending stump and kneaded eraser to smear or lighten areas to reflect the tone and texture of the organism’s body.

9) Instructors may want to emphasize “the process, not the product”; that is, students are to observe, interpret, and express anatomy and morphology, rather than focus on a beautiful product.

**Grading Rubric**

1) This assignment may be viewed as equivalent to a short writing assignment of 400-600 words. As such, it might represent approximately 5-10% of the total points used to determine a student’s grade.

2) Elements to consider in a grading rubric. Assume that the drawing assignment is worth 30 points in total.

| Element | Points | Comments |
| --- | --- | --- |
| Anatomy | 18 to 22 | As this drawing is to be realistic, the student is expected to accurately capture anatomical features in terms of appearance, placement on the body, and overall proportions. |
| Labels | 2 to 3 | Required elements may include: minimum number of labelled body parts (e.g., 5 or more), printed words are legible and of sufficient size, size and placement of words and label lines do not obscure or detract from the drawing. |
| Taxonomy | 2 to 3 | This drawing is an opportunity for the student to report an organism’s taxonomy. Depending on the course, common name and/or genus and species epithet may be sufficient, or the eight main taxonomic levels may be more appropriate (DKPCOFGS). |
| Effort | 2 to 3 | As this is a biology course, emphasis may be placed on rewarding a student’s effort, rather than aesthetics. |
| Aesthetics | 2 to 3 | Instructors may want to include this element to reward students that produce remarkable drawings, with the caveat that a drawing’s aesthetics or “beauty” can be rather subjective. |
